# Non-Abelian adiabatic geometric transformations in a cold strontium gas

**DOI:** 10.1038/s41467-018-05865-3

**Published:** 2018-09-04

**Authors:** F. Leroux, K. Pandey, R. Rehbi, F. Chevy, C. Miniatura, B. Grémaud, D. Wilkowski

**Affiliations:** 1 Centre for Quantum Technologies, National University of Singapore, Singapore, 117543 Singapore; 2 MajuLab, CNRS-UCA-SU-NUS-NTU International Joint Research Unit, Singapore, Singapore; 3 Laboratoire Kastler Brossel, ENS-PSL University, CNRS, Sorbonne Université, Collége de France, 24 Rue Lhomond, Paris, 75005 France; 4 Department of Physics, National University of Singapore, 2 Science Drive 3, Singapore, 117542 Singapore; 5 School of Physical and Mathematical Sciences, Nanyang Technological University, Singapore, 637371 Singapore

## Abstract

Topology, geometry, and gauge fields play key roles in quantum physics as exemplified by fundamental phenomena such as the Aharonov–Bohm effect, the integer quantum Hall effect, the spin Hall, and topological insulators. The concept of topological protection has also become a salient ingredient in many schemes for quantum information processing and fault-tolerant quantum computation. The physical properties of such systems crucially depend on the symmetry group of the underlying holonomy. Here, we study a laser-cooled gas of strontium atoms coupled to laser fields through a four-level resonant tripod scheme. By cycling the relative phases of the tripod beams, we realize non-Abelian SU(2) geometrical transformations acting on the dark states of the system and demonstrate their non-Abelian character. We also reveal how the gauge field imprinted on the atoms impact their internal state dynamics. It leads to a thermometry method based on the interferometric displacement of atoms in the tripod beams.

## Abstract (web summary)

The symmetry group and geometric phase of a system are responsible for many quantum properties related to non-trivial topology. Here the authors show non-Abelian geometric phase in laser-coupled ultracold strontium atoms by using a tripod scheme.

## Introduction

In 1984, Berry published the remarkable discovery that cyclic parallel transport of quantum states causes the appearance of geometrical phase factors^1^. His discovery, along with precursor works^2,3^, unified seemingly different phenomena within the framework of gauge theories^4,5^. This seminal work was rapidly generalized to non-adiabatic and noncyclic evolutions^5^ and, most saliently for our concern here, to degenerate states by Wilczek and Zee^6^. In this case, the underlying symmetry of the degenerate subspace leads to a non-Abelian gauge field structure. These early works on topology in quantum physics have opened up tremendous interest in condensed matter^7–11^ and more recently in ultracold gases^12–20^ and photonic devices^21–23^.

Moreover, it has been noted that geometrical qubits are resilient to certain noises, making them potential candidates for fault-tolerant quantum computing^24–27^. So far, beside some recent proposals^28,29^, experimental implementations have been performed for a two-qubit gate on NV centers in diamond^30^ and for a non-Abelian single-qubit gate in superconducting circuits^31^. These experiments were performed following a non-adiabatic protocol allowing for high-speed manipulation^29,32,33^. Recently, coherent control of ultracold spin-1 atoms confined in optical dipole traps was used to study the geometric phases associated with singular loops in a quantum system^34^. If non-adiabatic manipulations are promising methods for quantum computing, they prevent the study of external dynamic of quantum system in a non-Abelian gauge field, where non-trivial coupling occurs between the internal qubit state dynamics and the center-of-mass motion of the particle.

Here, we report on non-Abelian adiabatic geometric transformations implemented on a non-interacting cold fermionic gas of strontium-87 atoms by using a four-level resonant tripod scheme set on the $\,^1S_0,F_{\rm{g}} = 9/2 \rightarrow \,^3P_1,F_{\rm{e}} = 9/2$ intercombination line at *λ* = 689 nm (linewidth: $\Gamma = 2\pi \hskip1pt \times 7.5$ kHz). About 10^5^ atoms are loaded in a crossed optical dipole trap, optically pumped in the stretched Zeeman state $|F_{\rm{g}} = 9/2,m_{\rm{g}} = 9/2\rangle$ and Doppler cooled down to temperatures *T* ∼ 0.5 μK^35,36^, see Methods. A magnetic bias field isolates a particular tripod scheme in the excited and ground Zeeman substate manifolds. Our laser configuration consists of two co-propagating beams (with opposite circular polarizations) and a third linearly polarized beam orthogonal to the previous ones. These three coplanar coupling laser beams are set on resonance with their common excited state $|e\rangle = |F_{\rm{e}} = 9/2,m_{\rm{e}} = 7/2\rangle$.

## Results

### Dark states basis

**Fig. 1:**
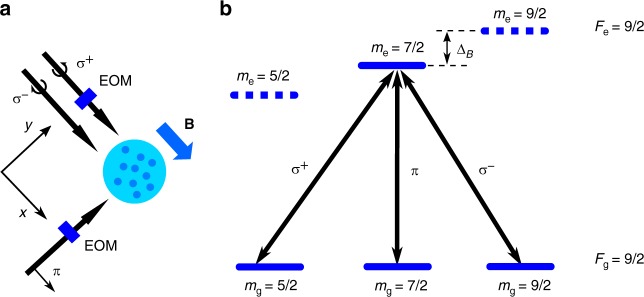
Tripod scheme. **a** Propagation directions of the laser beams and their polarizations along a magnetic bias field **B**. Two electro-optic modulators (EOM) are used to sweep the two independent relative phases of the laser beams. **b** Bare energy-level structure of the tripod scheme, implemented on the intercombination line of strontium-87. The magnetic bias field shifts consecutive excited levels by $\Delta _B \simeq 760\Gamma = 5.7{\kern 1pt}\,$MHz. It allows each tripod polarized laser beam to selectively address one of the magnetic transitions marked by the black arrows

For any value of the amplitude and phase of the laser beams, the effective Hilbert space defined by the four coupled bare levels contains two bright states and two degenerate dark states $|D_1\rangle$ and $|D_2\rangle$. These dark states do not couple to the excited state $|e\rangle$ and are thus protected from spontaneous emission decay by quantum interference. For equal Rabi transition frequencies, we conveniently choose

$$|D_1\rangle = \frac{{e^{ - i\Phi _{13}({\mathbf{r}})}|1\rangle - e^{ - i\Phi _{23}({\mathbf{r}})}|2\rangle }}{{\sqrt 2 }}$$

1 $$|D_2\rangle = \frac{{e^{ - i\Phi _{13}({\mathbf{r}})}|1\rangle + e^{ - i\Phi _{23}({\mathbf{r}})}|2\rangle - 2|3\rangle }}{{\sqrt 6 }},$$

where $|i\rangle \equiv |m_g = i + 3/2\rangle$ (*i* = 1,2,3). $\Phi _{ij} = \Phi _i - \Phi _j$, where the space-dependent laser phases read $\Phi _i({\mathbf{r}}) = {\mathbf{k}}_i \cdot {\mathbf{r}} + \vartheta _i$. **k**_*i*_ is the wavevector of the beam coupling state $|i\rangle$ to $|e\rangle$ and $\vartheta _i$ its phase at origin, see Fig. 1. To implement non-Abelian transformations on the system, the two independent offset phases tuned by the electro-optic modulators (EOM), shown in Fig. 1a, are $\phi _i = \vartheta _i - \vartheta _3$ (*I* = 1,2).

In a first set of experiments, we probe and quantify the thermal decoherence of the dark states induced by the finite temperature of our atomic sample. In a second set of experiments, we analyze the non-Abelian character of geometric transformations within the dark-state manifold. To do so, we consider a certain phase loop in the parameter space defined by the two relative phases *ϕ*_1_ and *ϕ*_2_ of the tripod lasers, and we compare the final populations of the internal atomic states when the cyclic sequence is performed, starting from two different initial points on the loop. In all experiments, we monitor the subsequent manipulation and evolution of the atomic system in the dark-state manifold by measuring the bare ground-state populations with a nuclear spin-sensitive shadow imaging technique, see Methods.

### Thermal decoherence

**Fig. 2:**
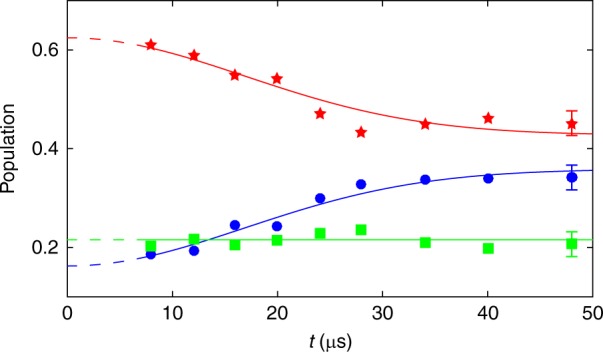
Ballistic expansion. Time evolution of the bare-state populations after the tripod ignition sequence (duration $t_0 \simeq 8{\kern 1pt}$ µs) is completed and laser beams have reached equal Rabi frequencies $\Omega = 2\pi \times 250{\kern 1pt}$ kHz. The blue circles, the green squares, and the red stars correspond to the populations $\bar P_1$, $\bar P_2$, and $\bar P_3$ with $|i\rangle \equiv |m_g = i + 3/2\rangle$ (*i* = 1, 2, 3), respectively. The error bars correspond to a 95% confidence interval. Solid lines: theoretical predictions given by Eq. (5). The temperature *T*, the initial and the final populations of each spin state are the fit parameters. The dashed lines, at early times, extrapolate the fits into the time window *t*_0_. We get a temperature $T = 0.5(1){\kern 1pt}$ µK meaning that the atoms do not move significantly during the dark-state preparation sequence since $\bar vt_0/\lambda \simeq 0.08$

Starting from state $|3\rangle$, we prepare the atoms in dark state $|D_2\rangle$ after a suitable adiabatic laser ignition sequence, see Methods. We assume that the atoms do not move significantly during the time duration of this sequence, see Fig. 2. Following refs. ^37,38^, the subsequent evolution of the atoms is described by the Hamiltonian

2 $$H = \frac{{({\hat {\mathbf p}} {1 \hskip-3.3pt 1} - {\mathbf{A}})^2}}{{2M}} + W$$

where $\hat{\mathbf p} = - i\hbar \nabla$ is the momentum operator, ${1 \hskip-3.3pt 1}$ is the identity operator in the internal dark-state manifold, *M* the atom mass, **A** the geometrical vector potential with matrix entries ${\mathbf{A}}_{jk} = i\hbar \langle D_j|\nabla D_k\rangle$ and *W* the geometrical scalar potential with matrix entries

3 $$W_{jk} = \frac{{\hbar ^2\left\langle {\nabla D_j|\nabla D_k} \right\rangle - \left( {{\mathrm{A}}^2} \right)_{jk}}}{{2M}} \cdot$$

With our laser geometry, **A**, **A**^2^, and *W* have the same matrix form, and are uniform and time-independent, see Methods. Thus, we can look for states in the form $|\psi \rangle \otimes |{\mathbf{p}}\rangle$ where **p** = *M***v** is the initial momentum of the atoms and $|\psi \rangle$ some combination of dark states. Denoting by *P*_0_(**v**) the initial atomic velocity distribution, we find that the population of state $|2\rangle$ remains constant while the two others display an out-of-phase oscillatory behavior at a velocity-dependent frequency $\omega _v = \frac{2}{3}[k(v_x - v_y) + 2\omega _R]$:

4 $$\begin{array}{*{20}{l}} {P_1({\mathbf{v}},t)} \hfill & { \hskip -09pt= \frac{{5P_0({\mathbf{v}})}}{{12}}\left( {1 - \frac{3}{5}{\rm{cos}}\omega _vt} \right),} \hfill \\ {P_2({\mathbf{v}},t)} \hfill & { \hskip -09pt= \frac{{P_0({\mathbf{v}})}}{6},} \hfill \\ {P_3({\mathbf{v}},t)} \hfill & { \hskip -09pt= \frac{{5P_0({\mathbf{v}})}}{{12}}\left( {1 + \frac{3}{5}{\rm{cos}}\omega _vt} \right),} \hfill \end{array}$$

where $\omega _R = \hbar k^2/(2M)$ is the recoil frequency and *k* = 2*π*/*λ* is the laser wavenumber. The frequency component proportional to $k(v_x - v_y)$ comes from the momentum-dependent coupling term ${\mathbf{A}} \cdot \hat{\mathbf p}/M$ in Eq. (2) (Doppler effect), whereas the other frequency component, proportional to *ω*_R_, comes from the scalar term ${\mathbf{A}}^2/(2M) + W$. With our laser configuration, light-assisted mechanical forces can only come from photon absorption and emission cycles between a pair of orthogonal laser beams. Such photon exchanges would induce a population change of state $|2\rangle$. Since no force is acting here on the center of mass of the atoms (the Abelian gauge field is uniform and can be gauged away), the population *P*_2_(**v**, *t*) must stay constant, as predicted by Eq. (4). Since photon absorption and emission cycles between the pair of co-propagating laser beams do not impart any net momentum transfer to the atoms, population transfer between states $|1\rangle$ and $|3\rangle$ is possible and *P*_1_(**v**, *t*) and *P*_3_(**v**, *t*) change in time, their sum being constant due to probability conservation.

Averaging over the Maxwellian velocity distribution of the atoms, the bare-state populations of the thermal gas read

$$\bar P_1(t) = \frac{5}{{12}} - \frac{1}{4}{\rm{cos}}\left( {\frac{4}{3}\omega _Rt} \right){\rm{exp}}\left[ { - \frac{4}{9}\left( {k\bar vt} \right)^2} \right],$$

$$\bar P_2(t) = \frac{1}{6},$$

5 $$\bar P_3(t) = \frac{5}{{12}} + \frac{1}{4}{\rm{cos}}\left( {\frac{4}{3}\omega _Rt} \right){\rm{exp}}\left[ { - \frac{4}{9}\left( {k\bar vt} \right)^2} \right],$$

where $\bar v = \sqrt {k_BT/M}$ is the thermal velocity of the gas at temperature *T*. We see that $\bar P_1$ and $\bar P_3$ converge to the same value at long times. This means that the thermal average breaks the tripod scheme into a Λ-scheme coupled to the two circularly polarized beams and a single leg coupled to the linearly polarized beam. As a consequence, quantum coherence partially survives the thermal average.

Our experimental results confirm this behavior even if $\bar P_1$ and $\bar P_3$ do not merge perfectly, see Fig. 2. This discrepancy can be lifted by introducing a 10% imbalance between the Rabi transition frequencies in our calculation. The population difference $\bar P_3 - \bar P_1$ measures in fact the Fourier transform of the velocity distribution along the diagonal direction $\hat{\mathbf x} - \hat{\mathbf y}$. It decays with a Gaussian envelope characterized by the time constant $\tau = 3/(2k\bar v)$, as predicted by Eq. (5). This interferometric thermometry is similar to some spectroscopic ones such as recoil-induced resonance^39,40^ or stimulated two photons transition^41,42^. From our measurements, we get *T* = 0.5(1) μK, $\tau \simeq 24\,{\kern 1pt}$μs and $\bar v \simeq 6.9{\kern 1pt}$ mm/s.

### Non-Abelian transformations

We now investigate the geometric non-Abelian unitary operator *U* acting on the dark-state manifold when the relative phases of the tripod beams are adiabatically swept along some closed loop *C* in parameter space. For a pinned atom $\left( {M \to \infty } \right)$, *U* is given by the loop integral along *C* of the 2 × 2 Mead–Berry 1-form $\omega \equiv [\omega _{jk}] \equiv [i\hbar \langle D_j|dD_k\rangle ]$

6 $$U = {\frak{P}}{\rm{exp}}\left( {\frac{i}{\hbar }{\oint}_C \omega } \right),$$

where ${\frak{P}}$ is the path-ordering operator^6^.

**Fig. 3:**
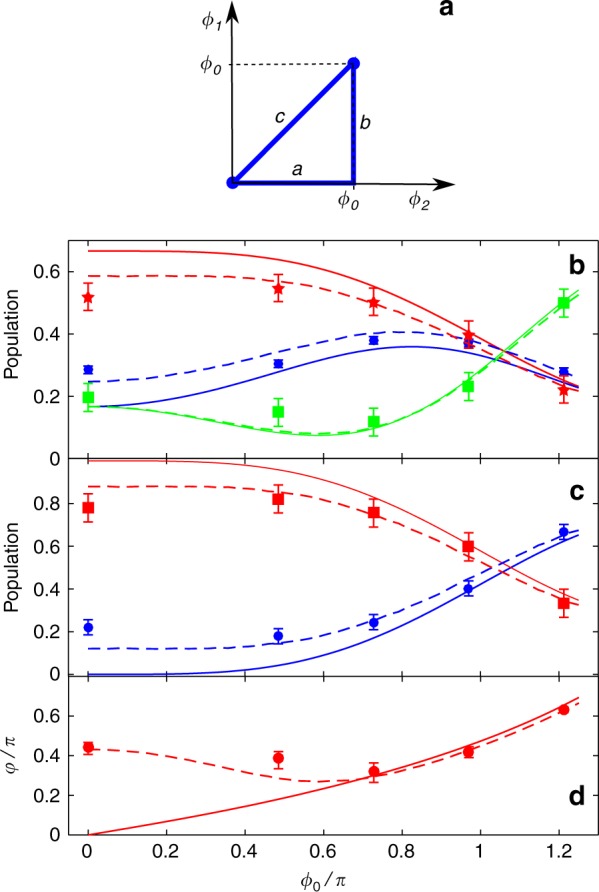
Geometric gate operation. **a** Phase loop in parameter space (*ϕ*_2_, *ϕ*_1_). *ϕ*_i_ (*i* = 1, 2) are the two independent offset phases tuned by the EOMs shown in Fig. 1a. We have performed two counterclockwise cycles: the first one is $a \to b \to c$ and starts from the origin, the second one is $c \to a \to b$ and starts from the upper corner. The loop is completed in 12 μs and its excursion is *ϕ*_0_. **b** Measured bare-state populations $\bar P_1$ (blue circles), $\bar P_2$ (green squares), and $\bar P_3$ (red stars) as a function of *ϕ*_0_ for the first cycle. The Rabi frequencies are $\Omega = 2\pi \times 450{\kern 1pt}$ kHz and *T* = 0.5 μK. Dark-state reconstruction as a function of *ϕ*_0_. **c** Population of $|D_1\rangle$ (blue circles) and $|D_2\rangle$ (red squares). **d** Azimuthal phase *φ*. The solid and dashed curves in **b**, **c**, and **d** are the theoretical predictions for a pinned atom and for a gas at temperature *T*, respectively. The error bars correspond to a 95% confidence interval

As before, the system is initially prepared in dark state $|D_2\rangle$. Then, starting from the origin, the phase loop is cycled counterclockwise, see Fig. 3a. Each segment is linearly swept in Δ*t* = 4 μs and the phase excursion is *ϕ*_0_. The total duration 3Δ*t* of the loop is thus less than the thermal decoherence time *τ* discussed above. In Fig. 3b, we plot the bare-state populations measured right after the phase loop as a function of *ϕ*_0_ and their comparison to theoretical predictions for pinned atoms and for atoms at finite temperature under the adiabatic assumption. This clearly shows that thermal effects are an important ingredient to reproduce the experimental results and that the adiabatic approximation is well justified, see Methods. Note that the mismatch with pinned atoms decreases with increasing *ϕ*_0_. This is because the thermal decoherence is quenched by the increasing geometrical coupling among the dark states when the sweep rate $\gamma = \phi _0/\Delta t \hskip1.5pt > \hskip1.5pt k\bar v,\omega _R$ (The thermal decoherence quenching can be quantified by the bare population distance $\Delta P = \sqrt {\mathop {\sum}\nolimits_{i = 1}^3 (P_i - P_{0i})^2}$, where $P_i$ and $P_{0i}$ are the experimental and pinned-atom populations. At $\varphi _0 = \pi$, we get $\Delta P = 0.04(5)$. This value increases when $\varphi _0$ decreases, reaching $\Delta P = 0.19(5)$ at $\varphi _0 = 0$). As a further approximation, we now disregard thermal decoherence and consider that the system after the phase loop is described by a pure quantum state $|\psi _{out}\rangle = \mathop {\sum}\nolimits_{j = 1,2} d_j|D_j\rangle$ (A pure state is denoted by a density matrix $\rho$ fulfilling Tr$\{ \rho ^2\} = 1$. For a finite-temperature gas, we find Tr$\{ \rho ^2\} = 0.95$ at $\varphi _0 = \pi$. This value decreases when $\varphi _0$ decreases, reaching Tr$\{ \rho ^2\} = 0.8$ at $\varphi _0 = 0$). As shown in Fig. 3b, c, one can easily extract the dark-state populations and the absolute value of the azimuthal angle $\phi = {\rm{Arg}}(d_2) - {\rm{Arg}}(d_1)$ from the measured bare-state populations, see Methods. When $\phi _0 \hskip1.5pt \gtrsim \hskip1.5pt 0.7\pi$, the values for *φ* match well with the prediction for a pinned atom confirming the quenching of thermal decoherence. At *ϕ*_0_ = *π*, the two dark-state populations are almost equal. In the language of the Bloch sphere representation, this corresponds to a rotation of the initial south pole state $|D_2\rangle$ to the equatorial plane.

**Fig. 4:**
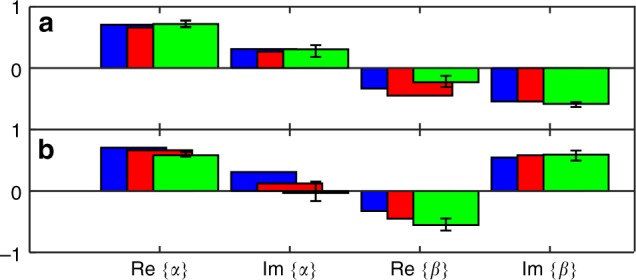
Unitary operators reconstruction. **a** Elements of operator *U* given by Eq. (7) for the phase loop $a \to b \to c$ at *ϕ*_0_ = *π*, see Fig. 3a. The blue, red, and green bars correspond to a pinned atom, a gas at temperature *T* = 0.5 μK, and the experimental data, respectively. **b** Same as **a**, but for the phase loop $c \to a \to b$. The error bars correspond to a 95% confidence interval

We now reconstruct the full geometric unitary operator *U* for *ϕ*_0_ = *π*. Up to an unobservable global phase, we write:

7 $$U = \left[ {\begin{array}{*{20}{c}} \alpha & \beta \\ { - \beta ^ \ast } & {\alpha ^ \ast } \end{array}} \right]$$

with $|\alpha |^2 + |\beta |^2 = 1$. The previous dark-state reconstruction, done after the phase loop applied on $|D_2\rangle$, gives access to $|\alpha |$, $|\beta |$ and ${\rm{Arg}}(\alpha ) - {\rm{Arg}}(\beta )$, see Methods. To obtain Arg(*α*) and Arg(*β*) and fully determine *U*, we start from a linear combination of dark states $|D_1\rangle$ and $|D_2\rangle$, perform the phase loop and process the new data. The results are shown in Fig. 4a and compared to the theoretical predictions for a pinned atom and a gas at finite temperature. The good agreement with our data validates the expected small impact of temperature for *ϕ*_0_ = *π*.

### Probing non-Abelianity

With the previous phase loop protocol, we have $U = U_cU_bU_a$, where *a*, *b*, and *c* label the edges of the loop, see Fig. 3a. To illustrate the non-commutative nature of the transformation group, we will cycle the phase loop counterclockwise starting from the upper corner. We then reconstruct the corresponding unitary operator $U^\prime = U_bU_aU_c$ like done for *U*. The results are depicted in Fig. 4b and show that *U* and *U*′ are indeed different, though unitarily related, confirming the sensitivity of these geometric transformations to path ordering. The Frobenius distance between the two unitaries is $D = \sqrt {2 - |{\mathrm{T}}r(U^\dagger U^\prime )|} = 1.27(25)$ and is in agreement with the theoretical result for a pinned atom (*D* = 1.09) and for a finite-temperature gas (*D* = 1.14). These values have to be contrasted with the maximum possible Frobenius distance *D* = 2.

## Discussion

Using a tripod scheme on strontium-87 atoms, we have implemented adiabatic geometric transformations acting on two degenerate dark states. This system realizes a universal geometric single-qubit gate. We have studied SU(2) transformations associated to laser beams phase loop sequences and shown their non-Abelian character. In contrast to recent works done in optical lattices^14–20^, our system realizes an artificial gauge field in continuous space. Depending on the laser field configuration, different manifestations of artificial gauge fields can be engineered such as spin–orbit coupling^38,43^, Zitterbewegung^38^, magnetic monopole^37^, or non-Abelian Aharomov–Bohm effect^43^ (see refs. ^44,45^ for reviews). A generalization to the SU(3) symmetry is also discussed in ref. ^46^. Some of these schemes might be difficult to implement in optical lattices. Gauge fields generated by optical fields come from a redistribution of photons among the different plane wave modes and involve momenta transfer comparable to the photon recoil. Observing mechanical effects of non-uniform or non-Abelian gauge fields would thus require atomic gases colder than the recoil temperature and thus cooling techniques beyond the mere Doppler cooling done here^47,48^. However, the gauge field is still driving the internal state dynamics regardless of the temperature of the gas provided the adiabatic condition is fulfilled. Noticeably, this internal state dynamics is still present when the gauge field is Abelian and uniform. It led us to an interferometric thermometry based on the Fourier transform of the velocity distribution of the gas.

## Methods

### Cold sample preparation and implementation of the tripod scheme

**Fig. 5:**
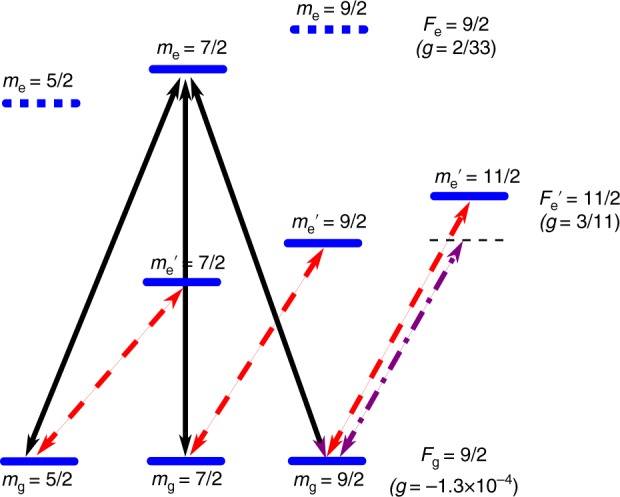
Energy levels and experimentally relevant transitions. A magnetic bias field *B* = 67 G lifts the degeneracy of the different Zeeman manifolds and allows to address each transition individually. The Landé factors *g* are indicated for each hyperfine level. The black arrows correspond to the tripod beams (see main text for more details). The dashed red arrows indicate the transitions used for the shadow spin-sensitive imaging system. The dash-dotted purple arrow is the red-detuned cooling transition used in the far off-resonant dipole trap

The cold gas is obtained by laser cooling on the $\,^1S_0 \to {\kern 1pt} \,^3P_1$ intercombination line at 689 nm (linewidth $\Gamma = 2\pi \times 7.5{\kern 1pt}$ kHz). Atoms are first laser cooled in a magneto-optical trap and then transferred into an ellipsoidal crossed optical dipole trap at 795 nm (trapping frequencies 150, 70, and 350 Hz), where they are held against gravity. Atoms are then optically pumped in the stretched $m_{\rm{g}} = F_{\rm{g}} = 9/2$ magnetic substate and subsequently Doppler cooled in the optical trap using the close $m_{\rm{g}} = F_{\rm{g}} = 9/2 \to m_{\rm{e}}^\prime = F_{\rm{e}}^\prime = 11/2$ transition, see Fig. 5. The atomic cloud contains about 10^5^ atoms at a temperature *T* = 0.5 μK (recoil temperature $T_R = \hbar \omega _R/k_{\rm{B}} \approx 0.23{\kern 1pt}$µK, where *k*_B_ is the Boltzmann constant). A magnetic field bias of *B* = 67 G is applied to lift the degeneracy of the Zeeman excited states. Because the Zeeman shift between levels in the excited manifold *F*_e_ = 9/2 is large, one can isolate a tripod scheme between three ground-state levels and a single excited state, namely $|e\rangle = |F_{\rm{e}} = 9/2,m_{\rm{e}} = 7/2\rangle$, as indicated in Fig. 5. The Zee man shift of the ground-state levels (Landé factor $g = - 1.3 \times 10^{ - 4}$) is weak (12 kHz) and is compensated by changing accordingly the frequencies of the three tripod laser beams. The lasers are finally tuned at resonance and their polarizations are chosen according to the electrical dipole transition selection rules. In practice, the two laser beams with right and left-circular polarizations, respectively, addressing the $m_{\rm{g}} = 5/2 \to m_{\rm{e}} = 7/2$ and $m_{\rm{g}} = 9/2 \to m_{\rm{e}} = 7/2$ transitions, are co-propagating. The laser beam with linear polarization, aligned with the magnetic bias field, addressing the $m_{\rm{g}} = 7/2 \to m_{\rm{e}} = 7/2$, is orthogonal to the circularly polarized beams, see Fig. 1b. The plane of the lasers is chosen orthogonal to the direction of gravity. The two independent laser offset phases *ϕ*_1_ and *ϕ*_2_ (see main text) can be tuned by using two electro-optic modulators.

### Adiabatic approximation

The two independent laser offset phases *ϕ*_1_ and *ϕ*_2_ are ramped from 0 to *ϕ*_0_ ≤ 1.2*π* at a constant rate γ during the sweep time Δ*t* = 4 μs. The AC-Stark shifts of the bright states is given by $\sqrt 3 \Omega = 2\pi \hskip1.5pt \times 780{\kern 1pt}$ kHz. Since $\gamma = \phi _0/\Delta t \le 2\pi \times 150{\kern 1pt}$ kHz, we have $\sqrt 3 \Omega /\gamma \ge 5.2$ and the adiabatic approximation is well justified.

### Initial dark-state preparation

Starting with atoms in the $|m_{\rm{g}} = 9/2\rangle$ stretched state, the tripod beams are turned on following two different sequences. The first sequence prepares dark state $|D_2\rangle$, see Eq. (1). More precisely, we first turn on the two laser beams connecting the empty bare states $|m_{\rm{g}} = 5/2\rangle$ and $|m_{\rm{g}} = 7/2\rangle$ to the excited state $|m_{\rm{e}} = 7/2\rangle$ and then adiabatically ramp on the last laser beam. This projects state $|m_{\rm{g}} = 9/2\rangle$ onto $|D_2\rangle$ with a fidelity of 95%. Since the bare-state $|m_{\rm{g}} = 9/2\rangle$ is only present in $|D_2\rangle$, our choice of basis in the dark-state manifold is well adapted to understand the dark-state preparation. A different ignition sequence is used to prepare a combination of dark states $|D_1\rangle$ and $|D_2\rangle$. By turning on sequentially abruptly the left-circular beam, and adiabatically the right-circular beam, we create a coherent (dark) superposition of the state $|m_{\rm{g}} = 5/2\rangle$ and $|m_{\rm{g}} = 9/2\rangle$. Finally, we turn on abruptly the linearly polarized beam and we expect to produce the linear combination $(|D_1\rangle + \sqrt 3 |D_2\rangle )/2$. In practice, a systematic phase rotation occurs once the last beam is turned on which adds an extra mixing among the dark states. Performing the bare-state population analysis, we find that this initial state corresponds in fact to $0.6|D_1\rangle + 0.8e^{i0.15\pi }|D_2\rangle$.

### Spin-sensitive imaging system

The bare-state populations in the ground-state are obtained with a nuclear spin-sensitive shadow imaging technique on the $F_{\rm{g}} = 9/2 \to F_{{\rm e}} {\hskip-3.5pt}{\,^\prime} = 11/2$ line, see Fig. 5. First, we measure the population of state $|m_{\rm{g}} = 9/2\rangle$ with a shadow laser tuned on the closed $m_{\rm{g}} = 9/2 \to m_{\rm{e}}\hskip-3.5pt \,^\prime = 11/2$ transition. Then, using the same atomic ensemble, we measure the population of state $|m_{\rm{g}} = 7/2\rangle$ by tuning the shadow laser on the $m_{\rm{g}} = 7/2 \to m_{\rm{e}} \hskip-3.5pt \,^\prime = 9/2$ transition. This transition is open but its large enough Clebsch–Gordan coefficient $\left( {\sqrt {9/11} \sim 0.9} \right)$ ensures a good coupling with the shadow laser. The population of state $|m_{\rm{g}} = 5/2\rangle$ is measured in the same way ($m_{\rm{g}} = 5/2 \to m_{\rm{e}} \hskip-3.5pt \,^\prime = 7/2$ open transition, its Clebsch–Gordan coefficient $\sqrt {36/55} \sim 0.8$ being still large enough). The shadow laser beam shines the atoms during 40 μs with an on-resonance saturation parameter *I*/*I*_s_ = 0.5 (saturation intensity $I_{\rm{s}} = 3 \hskip1.5pt {\upmu} {\mathrm{W}}/{\rm{cm}}^2$). With such values, the average number of ballistic photons scattered per atom is less than one and optical pumping can be safely ignored, ensuring an accurate measurement of the ground-state populations. To achieve a good statistics, the same experiment was repeated 100 times and the corresponding data averaged. The error bars on the bare-state populations correspond to a 95% confidence interval.

### Dark states and unitary matrix reconstruction

A state in the dark-state manifold takes the form $|\psi \rangle = \mathop {\sum}\nolimits_{j = 1,2} d_j{\kern 1pt} |D_j\rangle$ with $|d_1|^2$ and $|d_2|^2 = 1 - |d_1|^2$ the populations of states $|D_1\rangle$ and $|D_2\rangle$ and $\phi = {\mathrm{Arg}}(d_2) - {\mathrm{Arg}}(d_1)$ the azimuthal angle. Using Eq. (1), we immediately find

$$\begin{array}{*{20}{l}} {|d_2|} \hfill & { = \sqrt {3\bar P_3/2} ,} \hfill \\ {{\rm{cos}}\varphi } \hfill & { = (\bar P_1 - \bar P_2)/\sqrt {\bar P_3(2 - 3\bar P_3)} .} \hfill \end{array}$$

Do note that the normalization of $|\psi \rangle$ restricts the possible values of the $\bar P_i$ summing up to 1. The sign of *φ* is determined using the prediction of Eq. (6) for a pinned atom $\left( {M \to \infty } \right)$.

To reconstruct the unitary matrix *U*, as expressed in Eq. (7), we perform the phase loop sequence on two different initial dark states (their representative points on the Bloch sphere should not be opposite) and perform the dark-state reconstruction for each of them. The two phase terms in *U* are reconstructed up to a sign. As for the dark-state reconstruction, we rely on the prediction for a pinned atom to lift this sign ambiguity.

### Gauge fields and adiabatic Schrödinger equation

The time-dependent interaction operator for the resonant tripod scheme, in the rotating-wave approximation, has the following expression:

8 $$H(t) = \frac{{\hbar \Omega (\mathbf{r},t)}}{2}\mathop {\sum}\limits_{i = 1}^3 |e\rangle \langle i| + \mathrm{H.c.}$$

We assume here that the laser Rabi frequencies coupling the ground states $|i\rangle = |m_{\rm{g}} = i + 3/2\rangle$ to the excited state $|e\rangle = |m_{\rm{e}} = 7/2\rangle$ have all the same amplitude denoted by Ω. The time dependency comes from the cyclic ramping sequence of the two offset laser phases *ϕ*_*j*_ (*j* = 1, 2). Neglecting transitions outside the dark-state manifold (adiabatic approximation), the system is described by a quantum state $|\psi (\mathbf{r},t)\rangle = \Sigma _{j = 1,2}\Psi _{j}(\mathbf{r},t)|D_{j}(\mathbf{r},t)\rangle$, where $\Psi _{j}$ is the wave function of the center of mass of the atom in an internal state $|D_j\rangle$. In this basis, the adiabatic Schrödinger equation for the column vector $\underline \Psi = (\Psi _1,\Psi _2)^T$ reads:

9 $$i\hbar \dot{\underline {\Psi}} = \left[ {\frac{{\left(\hat {{\bf{p}}}{1 \hskip-3.4pt 1} - {\bf{A}}\right)^{2}}}{2M} + W - \omega _{t}} \right]\underline {\Psi},$$

where the dot denotes time derivative. The first two terms on the right-hand side describe the dynamics of an atom subjected to the synthetic gauge field. The last term $\omega _t \equiv [\omega _{jk}] \equiv [i\hbar \langle D_j|\dot D_k\rangle ]$ is due to the cyclic ramping sequence of the laser phases. Only this term remains for a pinned atom $\left( {M \to + \infty } \right)$, in which case one recovers Eq. (6). The general expressions of **A** and *W* are given in the main text. With equal and constant Rabi frequencies amplitude, and for the orientation of our laser beams, one finds:

$$\begin{array}{*{20}{l}} {\mathbf{A}} \hfill & { \hskip -09pt= \frac{{2\hbar \left({\mathbf{k}}_{\mathrm{2}}-\hskip1pt{\mathbf{k}}_{\mathrm{1}}\right)}}{{\mathrm{3}}}{\cal M},} \hfill \\ {\frac{{{\mathbf{A}}^2}}{{2M}}} \hfill & { \hskip -09pt= \frac{{8E_R}}{9}{\cal M},} \hfill \\ W \hfill & { \hskip -09pt= - \frac{{4E_R}}{9}{\cal M},} \hfill \end{array}$$

where $E_R = \hbar \omega _R = \hbar ^2k^2/(2M)$ is the recoil energy and **k**_*j*_ is the wavevector of laser beam *j* (see main text). As one can see, all these operators have the same matrix form. The matrix ${\cal M}$ reads:

10 $${\cal M} = \frac{{{1 \hskip-3.3pt 1} + \hskip1.5pt {\mathbf{s}} \cdot {\mathbf{\sigma }}}}{2} = \left( {\begin{array}{*{20}{c}} {3/4} & { - \sqrt 3 /4} \\ { - \sqrt 3 /4} & {1/4} \end{array}} \right),$$

and satisfies $\mathcal{M}^{\mathrm{2}} = \mathcal{M}$, its unit Bloch vector being ${\mathbf{s}} = ( - \sqrt 3 /2,0,1/2)$. As a consequence, all these operators can be diagonalized at once by the same transformation and amenable to the simple projector matrix form:

11 $${\cal M} \to {\cal M}_D = \left( {\begin{array}{*{20}{c}} 1 & 0 \\ 0 & 0 \end{array}} \right).$$

Because of our laser beams geometry, the vector potential **A** is in fact Abelian since its only non-zero matrix component is along **k**_2_ − **k**_1_.

In contrast, the operator *ω*_*t*_ has a different matrix form. Indeed, the two offset phases *ϕ*_*j*_ of the lasers (see main text) can be addressed at will. Following ref. ^44^, we get:

12 $$\omega _t = \frac{\hbar }{2}\left( {\begin{array}{*{20}{c}} {\dot \phi _1 + \dot \phi _2} & {(\dot \phi _1 - \dot \phi _2)/\sqrt 3 } \\ {(\dot \phi _1 - \dot \phi _2)/\sqrt 3 } & {(\dot \phi _1 + \dot \phi _2)/3} \end{array}} \right).$$

In particular, we note that *ω*_*t*_ leads to non-Abelian transformations. An immediate consequence is that, for a given phase loop in parameter space, the geometric unitary operator associated with a cycle of phase ramps depends on the starting point of the cycle on the loop. Different starting points lead to different, though unitarily related, non-commuting geometric unitary operators.

## Data Availability

The authors declare that the main data supporting the findings of this study are available within the article. Extra data are available from the corresponding author on request.
